# Design and synergistic effect of nano-sized epoxy-NiCo_2_O_4_ nanocomposites for anticorrosion applications†

**DOI:** 10.1039/d2ra01773c

**Published:** 2022-05-17

**Authors:** M. Swathika, Kshitij RB Singh, M. Mehala, Sadanand Pandey, Jay Singh, Ravindra Pratap Singh, Arunadevi Natarajan

**Affiliations:** Department of Chemistry, PSGR Krishnammal College for Women Coimbatore Tamil Nadu 641004 India arunadevi@psgrkcw.ac.in; Department of Chemistry, Institute of Science, Banaras Hindu University Varanasi Uttar Pradesh 221005 India; Department of Chemistry, College of Natural Science, Yeungnam University 280 Daehak-Ro Gyeongsan Gyeongbuk 38541 Republic of Korea; Department of Biotechnology, Indira Gandhi National Tribal University Amarkantak Madhya Pradesh 484887 India rpsnpl69@gmail.com ravindra.singh@igntu.ac.in

## Abstract

In the present work, we evaluated the corrosion inhibition properties of a ligand and mixed metal oxide nanocomposite. The ligand and mixed nickel–cobalt complex were synthesized using 1-naphthoic acid and aminoguanidine with the formulae [C_11_H_7_O_2_(CN_4_H_5_)(CN_4_H_6_)]·H_2_O and {Ni–Co[(CH_5_N_4_)_2_(C_11_H_7_O_2_)_2_]}·H_2_O, respectively. After their synthesis, physicochemical techniques such as CHNS analysis, infrared and UV-visible spectroscopy, thermal analysis, and X-ray diffraction (XRD) were employed to characterize both the synthesized ligand and nickel–cobalt complex. The metal oxide prepared from the decomposition of the metal complex was also characterized using several techniques to confirm its bonding and structure. In addition, the corrosion inhibition efficiency of the epoxy-ligand and epoxy-NiCo_2_O_4_ nanocomposite on mild steel (MS) in 3 M hydrochloric acid (HCl), 1.5 M sulfuric acid (H_2_SO_4_), and 0.5 M phosphoric acid (H_3_PO_4_) solution was examined and compared using weight loss measurements, Tafel plots, isotherms and electrochemical impedance spectroscopy (EIS). The results from the electrochemical studies disclosed that the epoxy coating of mixed metal oxides with 0.8 ppm concentration yielded excellent corrosion protection. The SEM images of mild steel and mild steel coated with epoxy-ligand/epoxy-NiCo_2_O_4_ in HCl confirmed the anti-corrosive behavior of the synthesized compounds. Hence, the as-prepared material can be a next-generation tool for sustainable anti-corrosive coatings.

## Abstract 2

This study reports the synthesis of nano-sized epoxy-NiCo_2_O_4_ nanocomposites and their anti-corrosive efficiency to attain sustainable development.

## Graphical abstract

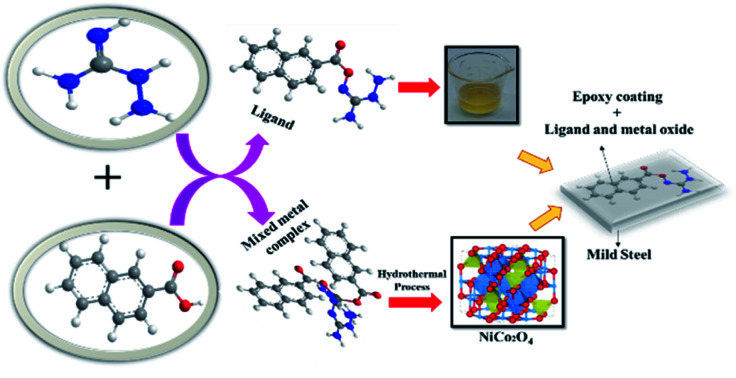

## 1. Introduction

Aminoguanidine is a hydrazine derivative and a bifunctional molecule, and the salts prepared from aminoguanidine are anticipated to resemble the hydrazine moiety. The nitrogen constituent and asymmetric bifunctional nature of aminoguanidine are responsible for its enhanced thermal and chemical reactivity. Thus, its salts are employed to prepare metal complexes with important physical properties such as ferroelectricity (pyroelectricity and piezoelectricity) and second harmonic generation.^1,2^ It is also an excellent chelating ligand and forms monodentate or bidentate complexes with metal ions. Due to the presence of carbon with four nitrogen atoms, aminoguanidine acts as an outstanding starting material in cyclization processes. Specifically, the four nitrogen atoms in aminoguanidine undergo coordination in various modes to form different types of complexes.^3,4^ However, in the free state, aminoguanidine is unstable, while in the solid state, it exists as a mono (+1) or di (+2) cation. In the solid form, its extreme nitrogen atom has hydrazine moiety in the sp^3^ hybridized state and the extra non-hydrogen atoms are in the sp^2^ hybridized state.^5,6^

Further, 1-naphthoic acid (1-naphthalene carboxylic acid) and its derivatives have attracted great attention from researchers because they possess an extensive range of biological and chemical applications in medicine, photochemicals, organic pigments, pesticides, photosensitive materials, dyes, cosmetic preparations, *etc.* Complexes of naphthalene derivatives have been examined since the 1960s, when 1-naphthoic acid was compiled from a planar dimer unit. It was mainly used to synthesize highly economical and effective herbicides, and it also acts as a plant growth regulator, thermal testimony material, and photosensitive object. Some materials prepared from 1-naphthoic acid have the resistivity of plasticizers and solvents, and these significant features led to the detailed spectral examination of 1-naphthoic acid, where one of the best examples is a naphthalene derivative, which is often used as a chelating agent.^7,8^

In recent years, epoxy resin has been used as an anti-corrosive resistance material in numerous applications such as electronics, automobiles, architectural pieces of equipment, and coatings for metallic materials, and as an adhesive.^9^ Mild steel possesses excellent mechanical properties such as corrosion resistivity, high tensile strength, and wear resistance.^10^ The metal oxide coating on mild steel has been examined using many metal oxides such as ZnO, SnO_2_, and TiO_2_. It was evident that the metal oxide coating enhanced the anti-corrosive property, and thus commonly used in anticorrosion paintings and coatings.^11^ In addition, metal oxides such as NiO and CoO show great chemical stability and photochemical and catalytic activity.^12^ Thus, many investigations have been carried out using mixed metal oxides such as Zn–Cr_2_O_3_–SiO_2_ and Zn–Al_2_O_3_–SiC, showing great corrosion-resistance properties.^13,14^

**Fig. 1:**
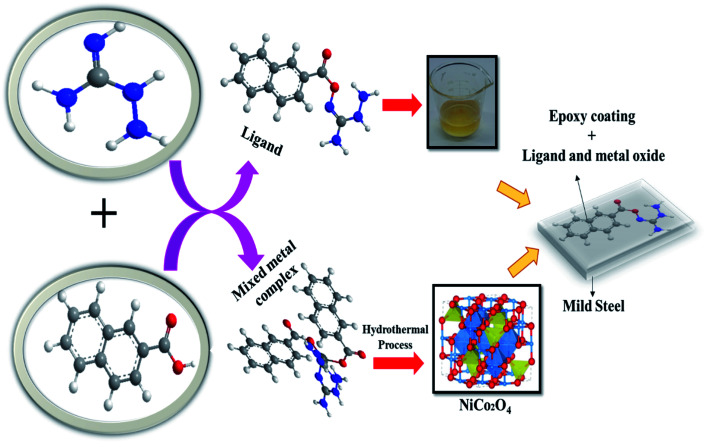
Overview of the present work.

Presently, to achieve sustainable development goals, researchers are focused on developing sustainable environmental protection and management methods. To achieve this, metal and mixed metal oxides have gained much attention due to their promising properties that make them suitable for various applications, namely anticorrosion, energy storage devices, sensors, and biosensors.^15–19^ Further, to achieve the objective of sustainable development, the grave concern of corrosion must be dealt urgently, owing to the fact that when metals are corroded they lose their original strength. Thus, the metal item has to be replaced, which requires the consumption of natural resources, resulting in devastating environmental degradation. Consequently, metal corrosion should be avoided by designing anti-corrosion materials to achieve sustainability. Hence, in the present work (Fig. 1), we synthesized ligand and mixed metal complexes of Ni(ii) and Co(ii) by utilizing the bulky 1-naphthoic acid as the core element to assemble with aminoguanidine to prepare a ligand and mixed metal complex. The mixed metal oxide prepared from the composite was layered externally on a metal surface. A fabricated steel plate was used to analyze its corrosion behavior in acidic solutions, namely, HCl, H_2_SO_4_, and H_3_PO_4_ acid solutions. The corrosion investigation of mild steel coated with epoxy-L and epoxy-NiCo_2_O_4_ nanocomposite was done using electrochemical techniques.

## 2. General procedure and materials

All chemicals and reagents employed in this study were procured from Sigma Aldrich and used as received. Further, 1-naphthoic acid, aminoguanidine bicarbonate, nickel nitrate, cobalt nitrate, and ethanol were procured from Aldrich. The other chemicals utilized for this study are readily available in the market. The synthetic procedure involved the synthesis of the ligand, mixed complexes, and mixed metal oxides in addition to the preparation of mild steel specimens.

### 2.1. Synthesis of ligand

The process for the preparation of the ligand involved mixing acid and base in the ratio of 1 : 2 by adding 0.1721 g of 1-naphthoic acid and 0.2722 g of aminoguanidine bicarbonate to 40 mL of Milli-Q water and heating in a water bath until they were completely dissolved. The resulting mixture was left undisturbed for 24, and the formed precipitate was washed with Milli-Q water and ethanol with centrifugation at 10 000 rpm two to three times, and then air-dried.

### 2.2. Synthesis of mixed metal complex

The mixed metal complex was prepared in the ratio of 1 (metal) : 1 (acid) : 2 (base). The ligand prepared in the above-mentioned step was added dropwise to 10 mL aqueous solution containing metals including Ni(ii) nitrate and Co(ii) nitrate (0.5 mmol/0.0913 g each) with constant stirring. The microcrystalline product was obtained instantly, and the solution was left undisturbed for one day. The crystalline product was filtered, splashed with water/alcohol several times at 10 000 rpm using a centrifuge machine, and desiccated at room temperature.

### 2.3. Synthesis of mixed metal oxide

The synthesized Ni–Co mixed metal complex was calcined at 800 °C in a muffle furnace and maintained at the same temperature for 8 h, where all the naphthoate and hydrazine moieties completely decomposed to form pure metal oxides, which were homogeneous and nano-size.

### 2.4. Preparation of mild steel specimens

Metal specimens were used as the working electrode. The chemical arrangement is presented in Table S1 (ESI†). The specimen coupons were shaped with dimensions of 3.1 cm × 1.0 cm × 0.05 cm with an exterior area of about 6.8 cm^2^. In the subsequent wiping process, the specimen was abraded using many grades of silicon carbide emery sheet. Then the metal specimen was splashed with tap water, followed by Milli-Q water, drained on a clean tissue paper after being immersed in acetone, and kept in a desiccator for further experiment.

### 2.5. Preparation of epoxy-ligand/epoxy-NiCo_2_O_4_ nanocomposite

The prepared nanoparticles (0.05 g, 0.4 g, and 0.8 g) were added to 2 mL of CHCl_3_ separately and vented in a sonicator for about 20–30 min. Then 2 mL of epoxy resin was mixed with the aforementioned solution dropwise with constant stirring for 3 h. Subsequently, 1 mL of hardener was mixed and agitated for 3 h. The final solution was collected, layered over the surface of the steel plates, and dehydrated at ambient temperature.

### 2.6. Physical measurements

The compositions of the ligand and mixed metal complexes were analyzed and confirmed by chemical and elemental analysis. A nitrogen test was carried out by incorporating the basic Lassaigne's test to confirm the binding of aminoguanidine in the metal composite and ligand. The presence of hydrazine in the ligand and metal composite was evaluated volumetrically by titrating the test solution *vs.* standard KIO_3_ (0.025 mol L^−1^) under Andrew's condition. A Vario-El III CHNS analyzer was used to determine the elements including carbon, oxygen, hydrogen, and nitrogen present in the ligand and metal complex. The percentage of metals present in the complex was also evaluated volumetrically by titrating with EDTA solution of 0.01 mol L^−1^. FT-IR spectroscopy was performed in the range of 4000–400 cm^−1^ on a PerkinElmer 597 spectrophotometer. UV-visible spectroscopy was performed on a Varian-Cary 5000 spectrophotometer in the wavelength range of 200–800 nm. TG-DTA (thermogravimetry-differential thermal analysis) was performed using an SDT Q600 V8.3 instrument from ambient temperature to 800 °C. X-ray powder diffraction patterns were recorded using a Philips X-ray diffractometer. The SEM and TEM and high-resolution (HR)-TEM analyses were carried out using a JEOL 6390 LA/OXFORD XMX-N and JEOL JEM 2100 HR-TEM, respectively.

### 2.7. Weight loss measurement

The weight of the metal plate was assessed accurately and it was immersed corrosive media with the help of glass hooks with and without inhibitor for almost 3 h at ambient temperature. Subsequently, the metal specimens were detached from the hook, cleaned with Milli-Q water, drained, and then weighed. The percentage inhibition efficiency (%IE) and corrosion rate were determined using eqn (1) and (2), respectively, where ‘*W*_b_’ stands for the weight loss of the blank (non-inhibitor) and ‘*W*_i_’ is the weight loss of the inhibitor. The same procedure was repeated using a temperature-controlled water bath at a higher temperature.1
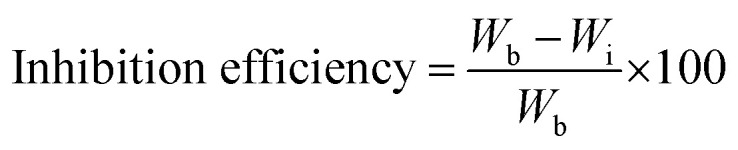
2
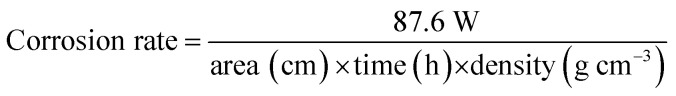


### 2.8. Electrochemical impedance (EIS)

EIS assessment was executed in a three-electrode system. Here, the reference electrode was a saturated calomel electrode (SCE); the platinum electrode was the counter electrode and steel sample as the working electrode. The electrochemical impedance assessment was performed on an Ivium CompactStat electrochemical unit. The % inhibition was evaluated by means of the charge transfer resistance (*R*_ct_) equation (eqn (3)), where ‘*R*_t(i)_’ and ‘*R*_t(b)_’ represent the electrochemical resistance for the inhibitor and blank, respectively.3
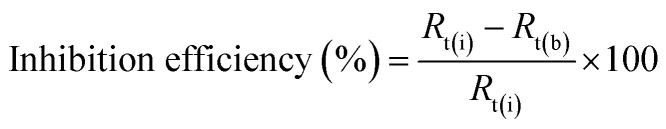


### 2.9. Tafel polarization

The Tafel plots were obtained by measuring both the anodic and cathodic curves at the scan rate of −200 to +200 mV at 1 mV s^−1^. The percentage polarization was estimated using the eqn (4), where ‘*I*_corr(b)_’ and ‘*I*_corr(i)_’ denote the corrosion current for the blank and inhibitor, respectively. Further, the polarization resistance (*R*_p_) was obtained from the linear slope, and the inhibition efficiency was also evaluated using eqn (5), where ‘*R*_p(b)_’ and ‘*R*_p(i)_’ represent the polarization resistance of the blank and inhibitor, respectively.4
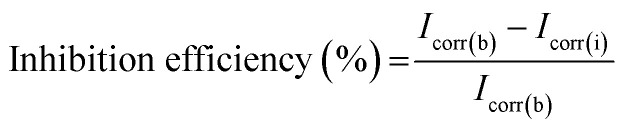
5
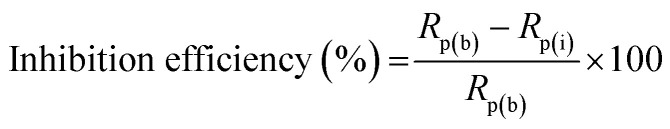


## 3. Findings

### 3.1. Analytical, thermal, optical, and morphological characterization

**Fig. 2:**
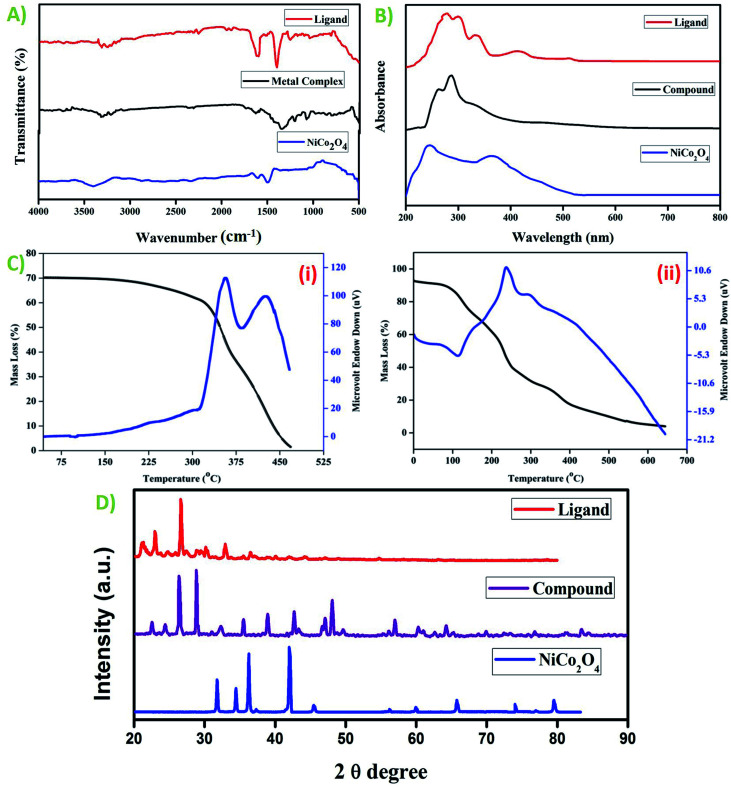
(A) FT-IR spectra of the ligand, metal complex, and NiCo_2_O_4_. (B) UV-visible spectra of the ligand, metal complex, and NiCo_2_O_4_. (C) Thermal analysis of (i) ligand and (ii) metal complex and (D) powder XRD patterns of ligand, metal complex, and NiCo_2_O_4_.

The composition of both the ligand and metal complex was confirmed from the analytical data. The molecular weight (MW), flash point, % calculated metal, hydrazine, and elements such as carbon, hydrogen, oxygen, and nitrogen present in the ligand and mixed metal complex are presented in Table 1. The chemical formula for the synthesized compounds was derived based on the elemental analysis. Table S2† shows the spectral data of the ligand and mixed metal complex. The ligand and complex show bands at the frequencies of 3316 cm^−1^ and 3321 cm^−1^, respectively, which substantiate the presence of water molecules in these compounds. The N–N stretching frequency of the ligand was detected at 1035 cm^−1^, which shifted to 1074 cm^−1^ for the complex. The asymmetric and symmetric stretching bands for the ligand were observed at 1616 cm^−1^ and 1398 cm^−1^, with Δ*ν* = *ν*_asym_ − *ν*_sym_ (average separation) of 218 cm^−1^. In the case of the metal complex, these bands were observed at 1631 cm^−1^ and 1348 cm^−1^, and the average separation was about 283 cm^−1^, which confirms the metal–carboxylic acid group linkage. The C=N stretching frequency of the ligand and metal complex was detected at 1258 cm^−1^ and 1203 cm^−1^, respectively. In addition, the bands 3244 cm^−1^ and 3211 cm^−1^ represent the N–H frequencies of the ligand and metal complex, respectively. The two characteristic vibrations of the M–O linkage of the Ni–Co metal complex were observed at 461 cm^−1^ and 504 cm^−1^, confirming the presence of two metal components in the complex.^20–24^ The two characteristic intense peaks at the lower frequency of 450 cm^−1^ and 502 cm^−1^ are attributed to the Ni–O and Co–O stretching vibrations in the metal oxide, and the double peaks at 1510 cm^−1^ and 1603 cm^−1^ are assigned to the characteristic nickel–cobalt oxide spinel composition formation. The wide peak at 3411 cm^−1^ confirms the presence of hydroxy groups, which may be due to physically adsorbed water molecules. The absence of bands between 900–1500 cm^−1^ and 1800–3000 cm^−1^ confirms the mainly carbon composite (C=O and C=H absorption peaks, respectively), as presented in Fig. 2A.

**Table 1:** Analytical and elemental data

Molecular formula	Color	Molecular weight	M.pt/°C	Analytical data (%)					
				Carbon found (calcd.)	Hydrogen found (calcd.)	Nitrogen found (calcd.)	Oxygen found (calcd.)	Hydrazine found (calcd.)	Metal found (calcd.)
[C_11_H_7_O_2_(CN_4_H_5_)(CN_4_H_6_)]·H_2_O – ligand	Orange	336.17	120	52.85 (52.87)	4.06 (4.07)	20.53 (20.55)	11.73 (11.74)	9.4 (9.2)	—
{Ni–Co[(CH_5_N_4_)_2_(C_11_H_7_O_2_)_2_]}·H_2_O – complex	Light brown	623.07	118	46.01 (46.22)	4.11 (4.17)	18.00 (17.97)	12.43 (12.83)	10.41 (10.37)	18.17 (18.74)

The UV-visible spectra of the synthesized ligand and complex in DMSO solvent were measured at room temperature. The UV spectral data is shown in Fig. 2B. The ligand exhibits an absorption band at 263 nm, which corresponds to the π → π* transition of the benzene moiety present in its acid part.^25^ The other peak at 286 nm corresponds to the π → π* transition of the aminoguanidine base group. Five absorption bands at 287, 302, 334, 426, and 480 nm were observed for the metal complex. The bands at 287 nm and 302 nm are attributed to the π → π* transitions of the ligand moiety, which are the ligand to metal charge transfer (LMCT) and localized transitions, respectively. The broader band at 334 nm corresponds to the ^1^A_1g_ → ^1^A_2g_ transition for the Ni(ii) ion, and the bands at 426 nm and 511 nm correspond to the ^1^A_1g_ → ^1^T_2g_ and ^1^A_1g_ → ^1^T_1g_ transitions, respectively for the Co(ii) metal ion.^26,27^ The absorption bands for the mixed metal oxide were observed at 243 nm for NiO and 364 nm for CoO. Two bands were observed because of the scattering from the nano metal oxide constituents of different sizes in the composite, resulting from the individual oxide phases.^28^

The TG-DTA graph of the ligand (Fig. 2C(i)) and complex (Fig. 2C(ii)) demonstrates a very slight endotherm at 99 °C in the DTA curve, which confirms the loss of water molecules in the ligand. Thus, it confirms the presence of coordinated water molecules. The exothermic shoulder peak from 313 °C to 446 °C is attributed to the decomposition of the carboxylate ion and the base moiety with a mass loss of almost 90%, leading to carbon residue as the final product. The metal complex showed three stages of decomposition. The endothermic peak at around 100 °C is owing to the elimination of an H_2_O molecule present in the complex, and consequently the formation of the Ni–Co[(CH_5_N_4_)_2_(C_11_H_7_O_2_)_2_] intermediate takes place. The exothermic peak at 241 °C is attributed to the decomposition of an acid moiety, which shows 60% mass loss. The other slight exotherm at 310 °C corresponds to the decomposition of the aminoguanidine base moiety. The final residue was the formation of the mixed metal oxide (eqn (6) and (7)).^29,30^6
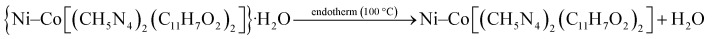
7
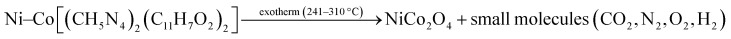


The X-ray diffraction (XRD) patterns of ligand, the complex, and NiCo_2_O_4_ exhibit intense peaks, indicating the crystalline nature of these compounds (shown in Fig. 2D). The significant difference between the XRD patterns of the ligand and the complex confirms the presence of metal ions in the metal complex.^31–34^ Further, the major diffraction peaks and 2*θ* values are indexed in Table S3.† Moreover, the obtained 2*θ* values were consistent with the Joint Committee on Powder Diffraction Standards (JCPDS) card number 73-1704 for NiCo_2_O_4_, confirming that the synthesized metal complex is nickel cobalt oxide and the intense peaks in the XRD pattern clearly show that the synthesized Ni–Co-oxide was highly pure.

**Fig. 3:**
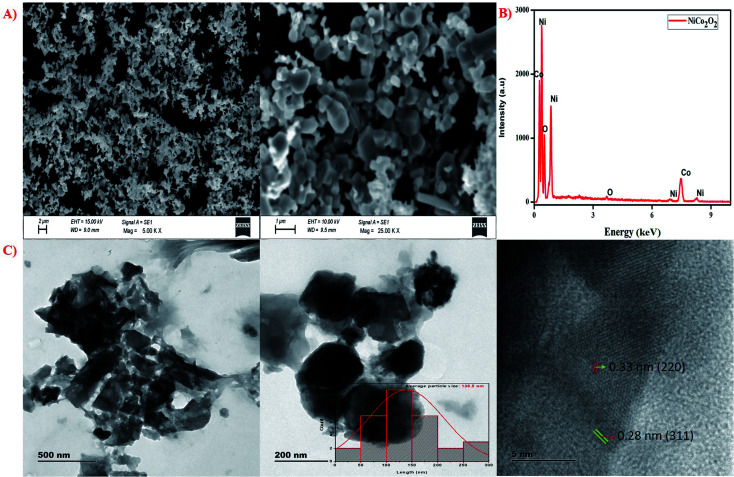
Nano NiCo_2_O_4_: (A) SEM images, (B) EDX analysis and (C) TEM and HR-TEM analysis.

The SEM images of nano NiCo_2_O_4_ are shown in Fig. 3A, confirming that the nano metal oxide had a homogeneous shape and size but demonstrated slight agglomeration owing to the presence of legends, given that the nano metal oxide was dispersed in the powder form to perform the SEM analysis. The SEM analysis revealed a spherical structure, which supports the glassy nature of the nano metal oxide. Further, the energy dispersive X-ray (EDX) analysis (Fig. 3B), which shows the presence of three elements, *i.e.*, Ni, Co, and O, also confirms the nanostructure of NiCo_2_O_4_. Furthermore, the TEM and HR-TEM analysis (Fig. 3C) revealed the shape and average particle size. The obtained average particle size from the quantitative analysis performed from the TEM micrograph was 138.5 nm (inset image in Fig. 3C, histogram fitted through Lorentzian function^18,35^), and the particles were spherical, which is consistent with the obtained SEM micrograph. Moreover, the HR-TEM image taken at 5 nm (Fig. 3C) reveals that the interference fringes with the *d*-spacing values of 0.33 nm and 0.28 nm correspond to the (220) and (311) planes of the NiCo_2_O_4_ crystal, respectively.

### 3.2. Electrochemical studies

#### 3.2.1. Weight loss method

Several corrosion parameters such as corrosion rate (mm per year), inhibition efficiency (% IE), and surface coverage (*θ*) were obtained using the weight-loss method and their values are shown in Table 2. The mass loss on mild steel was intentionally determined in 3 M HCl, 1.5 M H_2_SO_4_, and 0.5 M H_3_PO_4_, with an increase in inhibitor concentration and the absence of an inhibitor. This helped us determine the optimized concentration at which the strengthening of the resistive layer occurs. The inhibitor has strong adsorption due to its heteroatoms, which results in excellent surface coverage, thus reducing the metal deuteriation. The sequence of inhibitor efficacy was epoxy-NiCo_2_O_4_ > epoxy-L,^36^ which confirms the anti-corrosive nature of the chosen material.

**Table 2:** Corrosion parameters for various concentrations in mild steel by weight loss measurement at 303 K

Inhibitor		Concentration (ppm)	Weight loss (g)	Corrosion rate (mmpy)	Surface coverage (*θ*)	% IE
Epoxy-L	HCl	Blank	0.0678	37.04	—	
		0.05	0.026	14.20	0.6165	61.65
		0.4	0.018	9.83	0.7345	73.45
		0.8	0.010	5.46	0.8525	85.25
	H_2_SO_4_	Blank	0.148	80.86	—	—
		0.05	0.048	26.22	0.6757	67.57
		0.4	0.028	15.30	0.8108	81.08
		0.8	0.025	13.66	0.8311	83.11
	H_3_PO_4_	Blank	0.0772	42.18	—	
		0.05	0.0312	17.05	0.5959	59.59
		0.4	0.0228	12.46	0.7047	70.47
		0.8	0.020	10.93	0.7409	74.09
Epoxy-NiCo_2_O_4_	HCl	Blank	0.23	125.65	—	—
		0.05	0.056	30.59	0.7565	75.65
		0.4	0.019	10.38	0.9174	91.74
		0.8	0.001	0.55	0.9957	99.57
	H_2_SO_4_	Blank	0.0871	47.58	—	—
		0.05	0.0282	15.41	0.6762	67.62
		0.4	0.0101	5.52	0.8840	88.40
		0.8	0.005	2.73	0.9426	94.26
	H_3_PO_4_	Blank	0.0686	37.48	—	—
		0.05	0.0152	8.30	0.7784	77.84
		0.4	0.0101	5.52	0.8528	85.28
		0.8	0.009	4.92	0.8688	86.88

#### 3.2.2. Effect of temperature and activation parameters

**Fig. 4:**
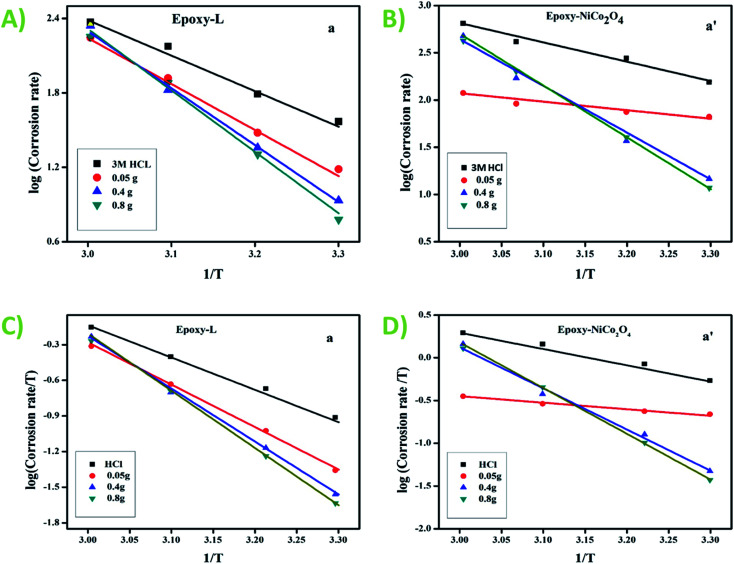
Corrosion rate in mild steel: Arrhenius plots with 3 M HCl (A) epoxy-L and (B) epoxy-NiCo_2_O_4_ and transition plot with HCl (C) epoxy-L and (D) epoxy-NiCo_2_O_4_.

The effect of temperature on the inhibition efficiency (IE) for the steel specimen was studied, and the results are presented in Table S4.† The experiment was carried out in three acids with and without inhibitors at 303, 313, and 333 K. The IE value for both inhibitors decreased when the temperature increased from 303 K to 333 K and showed a higher value for epoxy-NiCo_2_O_4_. This effect may be owing to the desorption mechanism, which takes place at high temperatures.^37^ The Arrhenius plots for HCl (Fig. 4A and B), H_2_SO_4_ (Fig. S1A and B†) and, H_3_PO_4_ (Fig. S1C and D†) and the transition plots for HCl (Fig. 4C and D), H_2_SO_4_ (Fig. S2A and B†), and H_3_PO_4_ (Fig. S2C and D†) were drawn using the data from the weight loss experiments, and the activation energy was calculated using eqn (8). Accordingly, it was inferred from Table 3 that the *E*_a_ value is directly proportional to the inhibitor concentration.^38^ Further, the enthalpy change (Δ*H*) shows a +ve (positive) value and increases with an increase in the inhibitor concentration. Overall, there was no association between concentration and Δ*H*, but a rise in concentration caused the film formation process to occur quicker. According to the *E*_a_, Δ*H*, and Δ*S* values, it was inferred that the energy barrier of the corrosion process was altered, and thus the reacting molecules will cross the barrier quickly, thereby speeding up the reaction.^39^8
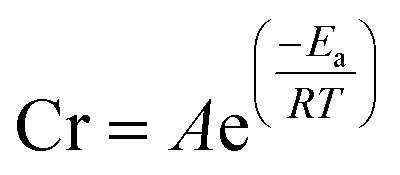


**Table 3:** Activation parameters for the corrosion of mild steel

Inhibition concentration	Epoxy-L			Epoxy-NiCo_2_O_4_		
Inhibitor	*E*_a_ (kJ)	ΔH° (kJ mol^−1^)	−ΔS° (kJ mol^−1^)	*E*_a_ (kJ)	ΔH° (kJ mol^−1^)	−ΔS° (kJ mol^−1^)
HCl	54.81	52.17	13.77	39.19	36.55	24.86
HCl + 0.05 g	70.99	68.35	59.55	17.28	14.64	104.82
HCl + 0.4 g	87.83	85.19	111.1	94.83	92.19	138.78
HCl + 0.8 g	94.81	100.56	92.47	105.04	62.23	14.95
H_2_SO_4_	32.53	29.89	52.25	46.15	43.51	11.40
H_2_SO_4_ + 0.05 g	59.72	57.08	28.42	70.86	68.22	60.40
H_2_SO_4_ + 0.4 g	74.97	72.33	75.07	104.45	101.81	164.09
H_2_SO_4_ + 0.8 g	86.32	57.70	78.62	113.57	85.04	155.84
H_3_PO_4_	32.97	30.33	57.21	61.78	59.14	39.99
H_3_PO_4_ + 0.05 g	57.47	54.83	16.14	97.91	95.27	148.83
H_3_PO_4_ + 0.4 g	61.78	59.14	28.03	108.05	105.41	178.36
H_3_PO_4_ + 0.8 g	80.48	64.59	38.71	179.95	62.30	209.64

#### 3.2.3. Adsorption isotherms

**Fig. 5:**
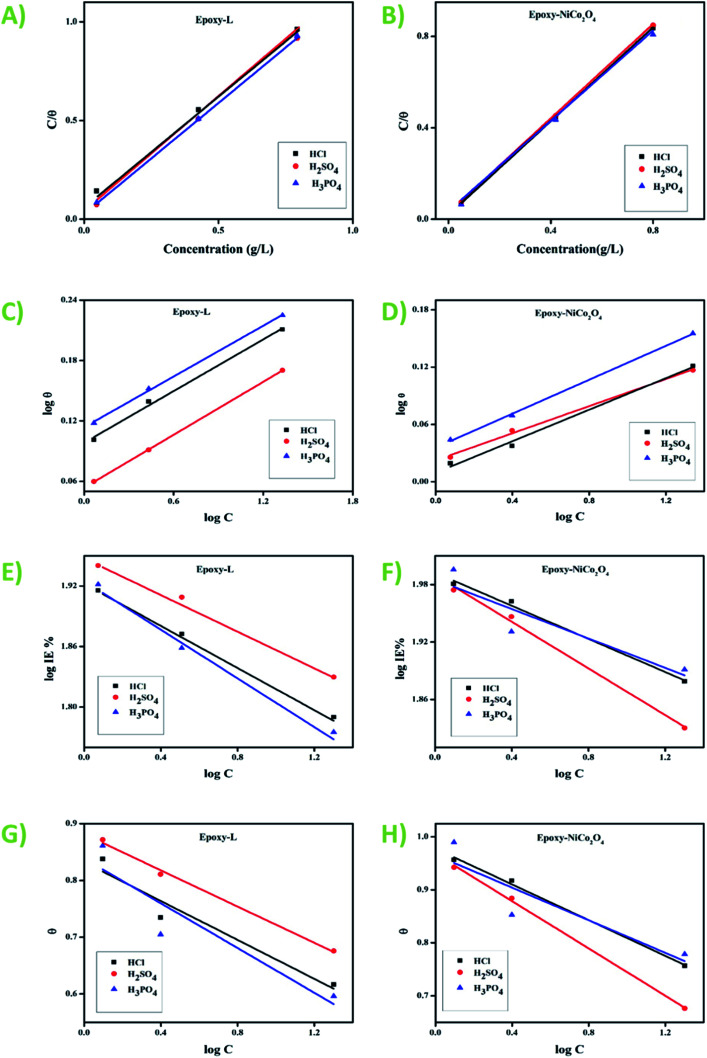
Langmuir, Freundlich, Frumkin, and Temkin isotherms of (A, C, E, and G) epoxy-L and (B, D, F, and H) epoxy-NiCo_2_O_4_, respectively.

Additional information about the interaction of epoxy-L and epoxy-NiCo_2_O_4_ with the metal surface was derived from the adsorption isotherm curves. A type of exchange takes place between the inhibitor and water molecules present on the surface of the specimen. Thus, to gain insight into the adsorption behavior of the synthesized inhibitors, several adsorption isotherms including the Langmuir, Freundlich, Frumkin, and Temkin isotherms were studied. In the case of the Temkin isotherm, it was evident that the plot fitted the experimental data well. The surface coverage value for different concentrations of the inhibitors was employed to find the best adsorption isotherm,^40^ as shown in Fig. 5. The correlation coefficient (*R*^2^) was evaluated and shown in Table S5.† Among the chosen acids, the best fit was gained with the Langmuir isotherm. Considering the correlation coefficient value, the accuracy of the Langmuir approach was seen clearly with good inhibition efficiency for the epoxy NiCo_2_O_4_ nanocomposite. Subsequently, we calculated the thermodynamic parameters for the metal oxide using the Langmuir adsorption isotherm. The standard free energy was calculated using eqn (9). The negative values of 
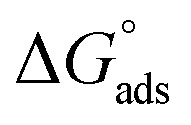
 indicate that the reaction occurs spontaneously on the surface. Generally, adsorption is considered to be physisorption or chemisorption, depending on the 
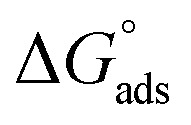
 value. According to Table S6,† the 
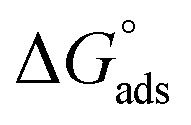
 values are in the range of −15.64 kJ mol^−1^ to −19.80 kJ mol^−1^ (<20 kJ mol^−1^), demonstrating that physical adsorption occurred on the steel surface.9
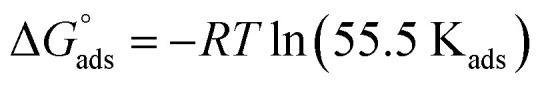


#### 3.2.4. Impedance spectroscopy

**Fig. 6:**
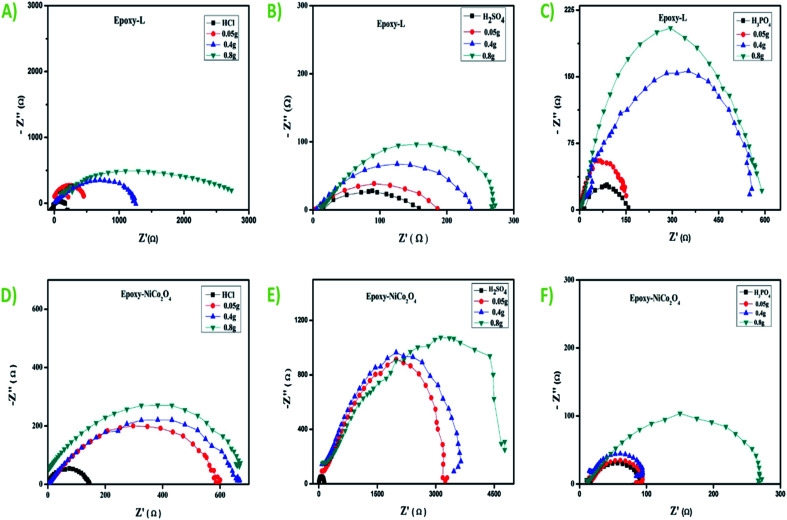
Nyquist diagram: epoxy-L with (A) HCl, (B) H_2_SO_4_, and (C) H_3_PO_4_ and epoxy-NiCo_2_O_4_ with (D) HCl, (E) H_2_SO_4_, and (F) H_3_PO_4_.

The Nyquist diagrams for the steel sample in different corrosive media including HCl (Fig. 6A and D), H_2_SO_4_ (Fig. 6B and E), and H_3_PO_4_ (Fig. 6C and F) were obtained and their semi-circles indicate that the charge transfer process is a controlled by a corrosion reaction. The impedance spectral response increased with an increase in the inhibitor concentration, and the *C*_dl_ and *R*_ct_ values are presented in Table 4. The increased *R*_ct_ values show the protection behavior of the inhibitor on mild steel, whereas the *C*_dl_ value decreases due to an increase in the protective layer thickness.^41^

**Table 4:** Electrochemical parameters at various concentrations

Concentration	Epoxy-L			Epoxy-NiCo_2_O_4_		
Inhibitor	*R*_ct_ (Ω cm^2^)	*C*_dl_ (μF cm^−2^)	Inhibition efficiency (%)	*R*_ct_ (Ω m^2^)	*C*_dl_ (μF cm^−2^)	Inhibition efficiency (%)
HCl	3.24	10.8		7.26	24.9	
HCl + 0.05 g	13.00	9.2	73.83	14.77	22.1	50.87
HCl + 0.4 g	20.46	7.8	83.36	21.77	18.5	66.65
HCl + 0.8 g	26.41	5.1	87.11	204.00	16.9	96.44
H_2_SO_4_	4.75	12.5		4.755	14.2	
H_2_SO_4_ + 0.05 g	8.39	10.2	43.40	79.02	12.7	93.98
H_2_SO_4_ + 0.4 g	17.17	9.7	72.33	108.34	9.7	95.61
H_2_SO_4_ + 0.8 g	24.84	8.7	80.87	114.64	7.7	95.85
H_3_PO_4_	21.15	17.3		45.153	29.3	
H_3_PO_4_ + 0.05 g	46.59	15.9	54.60	83.78	28.7	46.10
H_3_PO_4_ + 0.4 g	54.78	13.7	61.38	166.55	26.2	72.88
H_3_PO_4_ + 0.8 g	96.34	10.1	78.04	545.99	24.1	91.73

#### 3.2.5. Tafel polarization studies

**Fig. 7:**
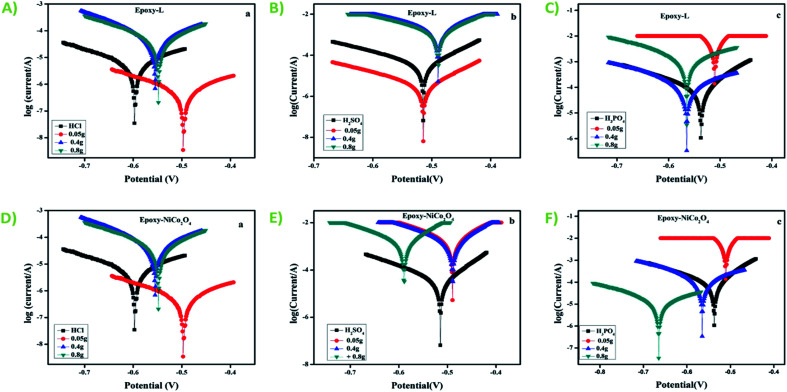
Polarization curves: epoxy-L with: (A) HCl, (B) H_2_SO_4_, and (C) H_3_PO_4_ and epoxy-NiCo_2_O_4_ with: (D) HCl, (E) H_2_SO_4_, and (F) H_3_PO_4_.

The potentiometric polarization studies were performed both in the presence and absence of inhibitor (Fig. 7). It was apparent from the graph that the *i*_corr_ value decreased with an increase in the inhibitor concentration, which may be due to the dissolution of the MS. The corrosion variables such as corrosion potential (*E*_corr_), corrosion current (*i*_corr_), and inhibition efficiency (IE%) attained from the Tafel plots are shown in Table S7.† There was no change in the cathodic or anodic slope with the addition of inhibitor, proposing a mixed-mode of inhibition process in mild steel.^42^ The inhibition efficiency of epoxy-NiCo_2_O_4_ inhibitor was higher than that of epoxy-L in 1 M HCl, proving that metal oxide adsorption capacity was higher.^43^

#### 3.2.6. Scanning electron microscopy and FTIR analysis

**Fig. 8:**
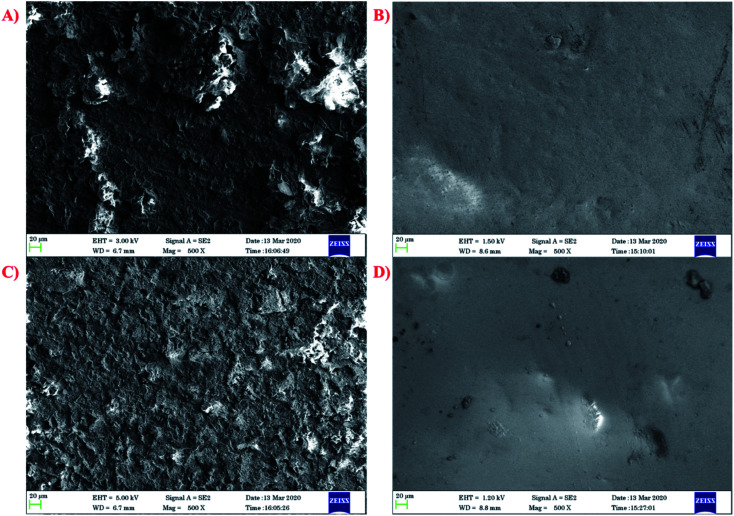
SEM micrographs of mild steel in HCl (A) and (C) without inhibitor and (B) and (D) with inhibitor.

The SEM micrographs (Fig. 8) reveal the changes that occurred on the external surface of the steel specimen both in the presence and absence of inhibitor. Fig. 8A and C display the damaged surface of the mild steel in the absence of the inhibitor and Fig. 8B and D confirm the development of a protecting layer in the presence of the inhibitor.^44^ Similarly, the coatings display a smooth surface on the mild steel with no superficial ruptures or cracks. The mixed metal oxide nanocomposite is distributed uniformly on the mild steel surface with few aggregates. This type of behavior is usually because while mixing nanoparticles in a viscous medium, there is the greater possibility that aggregation will occur.^45^

**Fig. 9:**
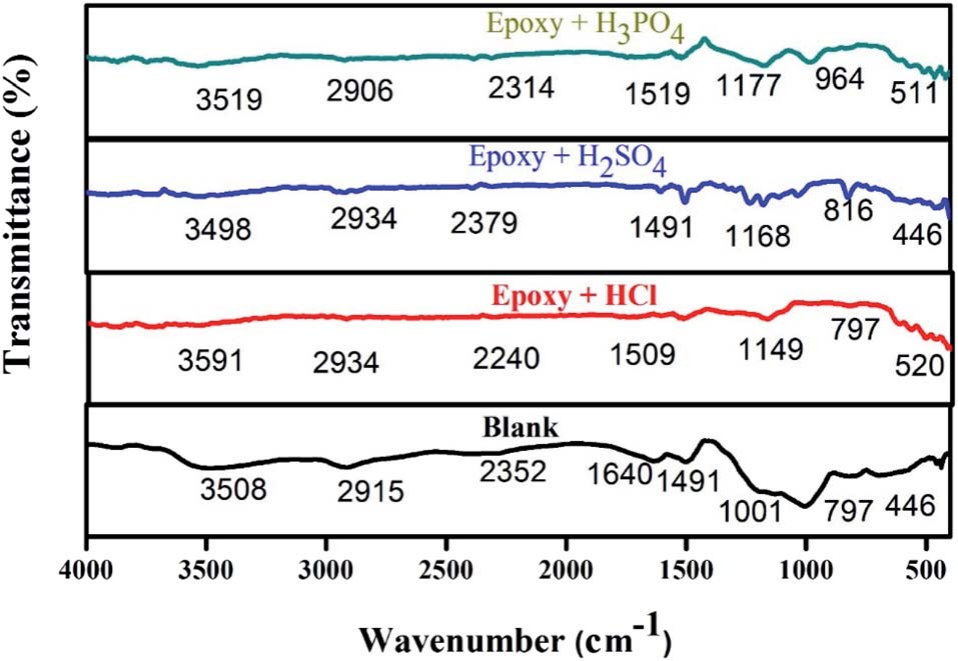
Infrared spectra of steel in different acids (HCl, H_2_SO_4_, and H_3_PO_4_).

The protecting film was observed on the metal surface using FT-IR analysis. The spectra of epoxy-L/NiCo_2_O_4_ are presented in Fig. 9. The wide peak detected at 3508–3525 cm^−1^ is attributed to the stretching frequency of the –NH moiety. The peaks at 2915, 2400, 2397, and 2906 cm^−1^ represent the C–H stretching. In addition, bands located at 612 and 850 cm^−1^ correspond to the bending vibration of the CH bond in the aromatic group. The peak at 446 cm^−1^ confirms the bond between the metal and oxygen. Hence, based on the FT-IR spectra, it can be concluded that the inhibitors formed active barriers on the metal surfaces.^46^

## 4. Proposed mechanism

**Fig. 10:**
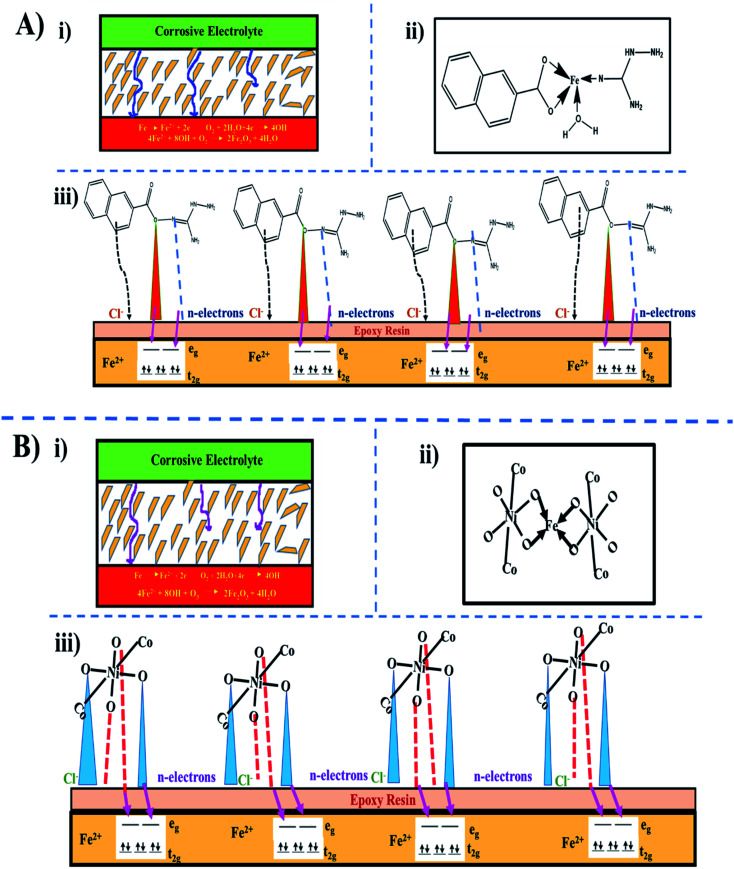
Proposed mechanism for inhibition protection: (A) epoxy-ligand and (B) epoxy-NiCo_2_O_4_, where (i) movement of corrosive electrolyte in epoxy-coated mild steel, (ii) linkage of inhibitor with Fe^2+^ ions, and (iii) electron transfer from inhibitor to mild steel.

The inhibition efficiency of mild steel depends on factors such as the functional groups, heteroatoms, and molecular structure of the inhibitor. If the bonding moiety contains carbon, hydrogen, and nitrogen atoms, these molecules become more hydrophobic in acidic media, which boosts the efficacy of inhibition. The mechanism of inhibition involves the interaction of the π-electrons from the naphthoic acid molecule and lone pair of electrons from all the donor heteroatoms to the vacant d-orbital of the acceptor iron molecule with the development of a stable film with uniform thickness.^47^ In our target molecule, the presence of three oxygen atoms, eight nitrogen atoms, and aromatic rings with conjugated double bonds increases the corrosion resistance. The cracks and minute holes formed by the evaporation of the solvents serve as canals for corrosive media and diminish the barrier activity. In terms of epoxy-NiCo_2_O_4_, the corrosive media reached the steel surface in a shorter duration compared to epoxy-ligand (Fig. 10A and B).^48^

## 5. Conclusion

Ni–Co complexes of 1-naphthoic acid with aminoguanidine and ligand molar ratio of 1 : 2 were synthesized and characterized. Different spectroscopy techniques including infrared and absorption spectroscopy, thermal analysis, and powder XRD diffraction were employed to characterize the ligand and metal composite, confirming the linkage of acid and base moiety with the metal ions. In the FT-IR analysis, the two different bands of M–O showed the linkage of the two metal ions in the composite. The ligand and metal complex were stable up to 98 °C and showed an endotherm, confirming the presence of one water molecule in the ligand moiety. Further, the decomposition of the metal composite resulted in the formation of NiCo_2_O_4_ as the end product. Epoxy-ligand/NiCo_2_O_4_ was examined for its anti-corrosive property using mild steel in three different types of acids including HCl, H_2_SO_4,_ and H_3_PO_4_ at different concentrations. The shielding effectiveness was improved with an increase in the concentration of epoxy-L/NiCo_2_O_4_. The superlative fit obtained in the Langmuir adsorption isotherm evidenced the excellent link between epoxy-NiCo_2_O_4_ and the exterior surface of the chosen mild steel specimen with a physisorption process. The Tafel plot result confirmed the mixed-mode corrosion protection behavior. The protection efficiency showed that epoxy-NiCo_2_O_4_ was a better inhibitor than epoxy-L in 3 M hydrochloric acid. The anti-corrosion layer formed by the inhibitors had outstanding barrier potential by hindering the exchange of H_2_O, O_2_ and corrosive particles. Thus, the synthesized mixed metal complex can be utilized in numerous industries for corrosion protection, which will be beneficial for achieving sustainability.

## Author contributions

M. S. and M. M. contributed to the data curation, investigation, resources, validation, and original draft writing. K. R. B. S. contributed to the conceptualization, data curation, investigation, resources, validation, writing the original draft, and reviewing & editing the manuscript draft. S. P. and J. S. contributed to the supervision, visualization, original draft writing, and manuscript draft editing. N. A. and R. P. S. contributed to the conceptualization, data curation, validation, project administration, supervision, writing the original draft, and reviewing & editing the manuscript draft.

## Conflicts of interest

Authors declare no conflict of interest for this work.

## Supplementary Material

RA-012-D2RA01773C-s001
