# Supplementary material for: Design and synergistic effect of nano-sized epoxy-NiCo2O4 nanocomposites for anticorrosion applications
Source: RSC Adv. 2022 May 17;12(23):14888–901. doi: 10.1039/d2ra01773c (PMC9112074; doi:10.1039/d2ra01773c)
Supplement: RA-012-D2RA01773C-s001 [file RA-012-D2RA01773C-s001.pdf]

Electronic Supplementary Material (ESI) for RSC Advances.
This journal is © The Royal Society of Chemistry 2022

Design and synergistic effect of nano-sized epoxy-$NiCo_2O_4$ nanocomposites for anticorrosion applications

M. Swathika[1], Kshitij RB Singh[2], M. Mehala[1], Sadanand Pandey[3], Jay Singh[2], Ravindra Pratap Singh[*,4], Arunadevi Natarajan[*,1]

[1]Department of Chemistry, PSGR Krishnammal College for Women, Coimbatore, Tamil Nadu (641004), India

[2]Department of Chemistry, Institute of Science, Banaras Hindu University, Varanasi, Uttar Pradesh (221005), India

[3]Department of Chemistry, College of Natural Science, Yeungnam University, 280 Daehak-Ro, Gyeongsan, Gyeongbuk (38541), Republic of Korea

[4]Department of Biotechnology, Indira Gandhi National Tribal University, Amarkantak, Madhya Pradesh (484887), India

[*]**Corresponding Authors**

Ravindra Pratap Singh (rpsnpl69@gmail.com, ravindra.singh@igntu.ac.in) and **N. Arunadevi** (arunadevi@psgrkcw.ac.in).

**Table S1** *Analytical chemical composition of mild steel (Wt %)*									
**Metal**	**Fe**	**C**	**P**	**Mn**	**Si**	**Cr**	**Mo**	**Al**	**S**
**Compositions**	Rem.	0.078	0.026	0.019	0.004	0.02	0.0009	0.004	0.02

**Table S2** *IR data of ligand and complex*		
**Assignments (cm^{-1})**	**Ligand (cm^{-1})**	**Complex (cm^{-1})**
ν (-OH)	3316	3321
ν (N-N)	1035	1074
ν_{asym} (C=O)	1616	1631
ν_{asym} (C=O)	1398	1348
ν_{asy} - ν_{sym}	218	283
ν (C=N)	1258	1203
ν (-NH)	3244	3211
ν (M-O)	-	461<br>504

Table S3 *XRD data of metal oxide*

2θ degree	hkl values
31.77	111 - NiO
34.53	220 - CoO
36.26	311 - CoO
37.36	222 - CoO
42.02	200 - NiO
45.44	400 - CoO
56.21	422 - CoO
60.01	511 - CoO
65.77	440 - CoO
74.09	621 -NiO
77.04	533 - CoO
79.48	622 - NiO

Table S4 *Corrosion parameters of mild steel solution in presence and absence of epoxy resin- Ligand/NiO-CoO at different temperatures (303±K - 333±K)*

Inhibition concentration	Temperature (K)	Epoxy-L			Epoxy -$NiCo_2O_4$		
		Weight loss (g)	Corrosion Rate (mm/year)	% IE	Weight loss (g)	Corrosion Rate (mm/year)	% IE
HCl	303	0.0678	37.04	-	230	125.65	-
	313	0.0995	54.36	-	651	355.66	-
	323	0.274	149.69	-	854	466.56	-
	333	0.428	233.83	-	979	534.85	-
HCl+0.05g	303	0.018	9.83	73.45	0.019	10.38	91.74
	313	0.037	20.21	62.81	0.134	73.21	79.42
	323	0.122	66.65	55.47	0.322	175.92	62.30
	333	0.401	219.08	6.31	0.598	326.70	38.92
HCl+0.4g	303	0.011	6.01	83.78	0.011	6.01	95.22
	313	0.047	25.68	52.76	0.178	97.25	72.66
	323	0.139	75.94	49.27	0.388	211.97	54.57
	333	0.328	179.19	23.36	0.524	286.27	46.48
HCl+0.8g	303	0.028	15.30	58.70	0.056	30.59	75.65
	313	0.048	26.22	51.76	0.278	151.88	57.30
	323	0.152	83.04	44.53	0.472	257.87	44.73
	333	0.322	175.92	24.77	0.0871	47.58	91.10
H_2SO_4-	303	0.148	80.86	-	0.0871	47.58	-
	313	0.238	130.03	-	0.189	103.26	-
	323	0.341	186.30	-	0.269	146.96	-
	333	0.477	260.60	-	0.483	263.88	-
H_2SO_4+0.05g	303	0.019	10.38	87.16	0.0101	5.52	88.40
	313	0.109	59.55	54.20	0.0558	30.48	70.48
	323	0.278	151.88	18.48	0.187	102.16	30.48

	333	0.418	228.36	12.37	0.421	230.00	12.84
**H_2SO_4+0.4g**	303	0.028	15.30	81.08	0.0282	15.41	67.62
	313	0.099	54.09	58.40	0.074	40.43	60.85
	323	0.252	137.67	26.10	0.156	85.23	42.01
	333	0.397	216.89	16.77	0.367	200.50	24.02
**H_2SO_4+0.8g**	303	0.048	26.22	67.57	0.005	2.73	94.26
	313	0.112	61.19	52.94	0.073	39.88	61.38
	323	0.254	138.77	25.51	0.158	86.32	41.26
	333	0.389	212.52	18.45	0.339	185.20	29.81
**H_3PO_4**	303	0.0772	42.18	-	0.0686	37.48	-
	313	0.0921	50.32	-	0.297	162.26	-
	323	0.157	85.77	-	0.439	239.84	-
	333	0.241	131.66	-	0.687	375.33	-
**H_3PO_4+0.05g**	303	0.0312	17.05	59.59	0.0007	0.38	98.98
	313	0.0487	26.61	47.12	0.137	74.85	53.87
	323	0.129	70.48	17.83	0.321	175.37	26.88
	333	0.222	121.28	7.88	0.611	333.80	11.06
**H_3PO_4+0.4g**	303	0.011	6.01	85.75	0.0152	8.30	77.84
	313	0.069	37.70	25.08	0.187	102.16	37.04
	323	0.127	69.38	19.11	0.354	193.40	19.36
	333	0.214	116.91	11.20	0.577	315.23	16.01
**H_3PO_4+0.8g**	303	0.0228	12.46	70.47	0.0101	5.52	85.28
	313	0.042	22.95	54.40	0.123	67.20	58.59
	323	0.102	55.73	35.03	0.267	145.87	39.18
	333	0.198	108.17	17.84	0.551	301.03	19.80

Table S5 *The correlation coefficient (R^2) value obtained from adsorption isotherm with epoxy resin-Ligand/NiO-CoO*

**Compound Inhibitor**		**Adsorption Isotherm**			
		**Langmuir**	**Freundlich**	**Frumkin**	**Temkin**
**Epoxy-L**	HCl	0.98	0.98	0.92	0.88
	H_2SO_4	0.97	0.98	0.99	0.91
	H_3PO_4	0.98	0.97	0.78	0.71
**Epoxy -$NiCo_2O_4$**	HCl	0.99	0.99	0.89	0.97
	H_2SO_4	0.99	0.98	0.88	0.98
	H_3PO_4	0.99	0.97	0.66	0.68

Table S6 *Thermodynamic parameters obtained for the epoxy resin-Ligand/NiO-CoO*

**Inhibitor**	**Compound**	**Langmuir**
		**ΔG°_{ads}(kJ/mol)**
**HCl**	**Epoxy-L**	-19.80
	**Epoxy -$NiCo_2O_4$**	-17.17
**H_2SO_4**	**Epoxy-L**	-18.16
	**Epoxy -$NiCo_2O_4$**	-18.35
**H_3PO_4**	**Epoxy-L**	-18.55
	**Epoxy -$NiCo_2O_4$**	-15.64

Table S7 *Tafel polarization at various concentrations*

Inhibitor	Epoxy-L					Epoxy -$NiCo_2O_4$				
	Tafelslopes (mV/dec)		E_{corr} (mV)	I_{corr} (μA/cm^2)	IE (%)	Tafelslopes (mV/dec)		E_{corr} (mV)	I_{corr} (μA/cm^2)	IE (%)
	b_a	b_c				b_a	b_c			
**HCl**	222	135	-567	347		315	132	-642	712	
**HCl+0.05g**	192	122	-551	291	33.2	219	127	-539	857	60.8
**HCl +0.4g**	182	109	-510	129	79.8	142	119	-512	522	80.2
**HCl+0.8g**	147	101	-430	119	89.3	139	101	-403	119	90.8
**H_2SO_4**	212	151	-555	254		298	122	-623	592	
**H_2SO_4+0.05g**	168	136	-452	247	30.1	251	115	-521	397	56.8
**H_2SO_4+0.4g**	149	119	-439	144	69.2	174	105	-422	254	79.2
**H_2SO_4+0.8g**	131	95	-413	124	77.8	160	87	-402	111	82.7
**H_3PO_4**	398	197	-512	423		435	169	-498	687	
**H_3PO_4+0.05g**	221	183	-417	328	20.9	357	138	-439	491	40.8
**H_3PO_4+0.4g**	210	150	-302	219	58.6	258	118	-401	357	60.5
**H_3PO_4+0.8g**	100	117	-221	123	60.3	189	109	-312	216	75.1

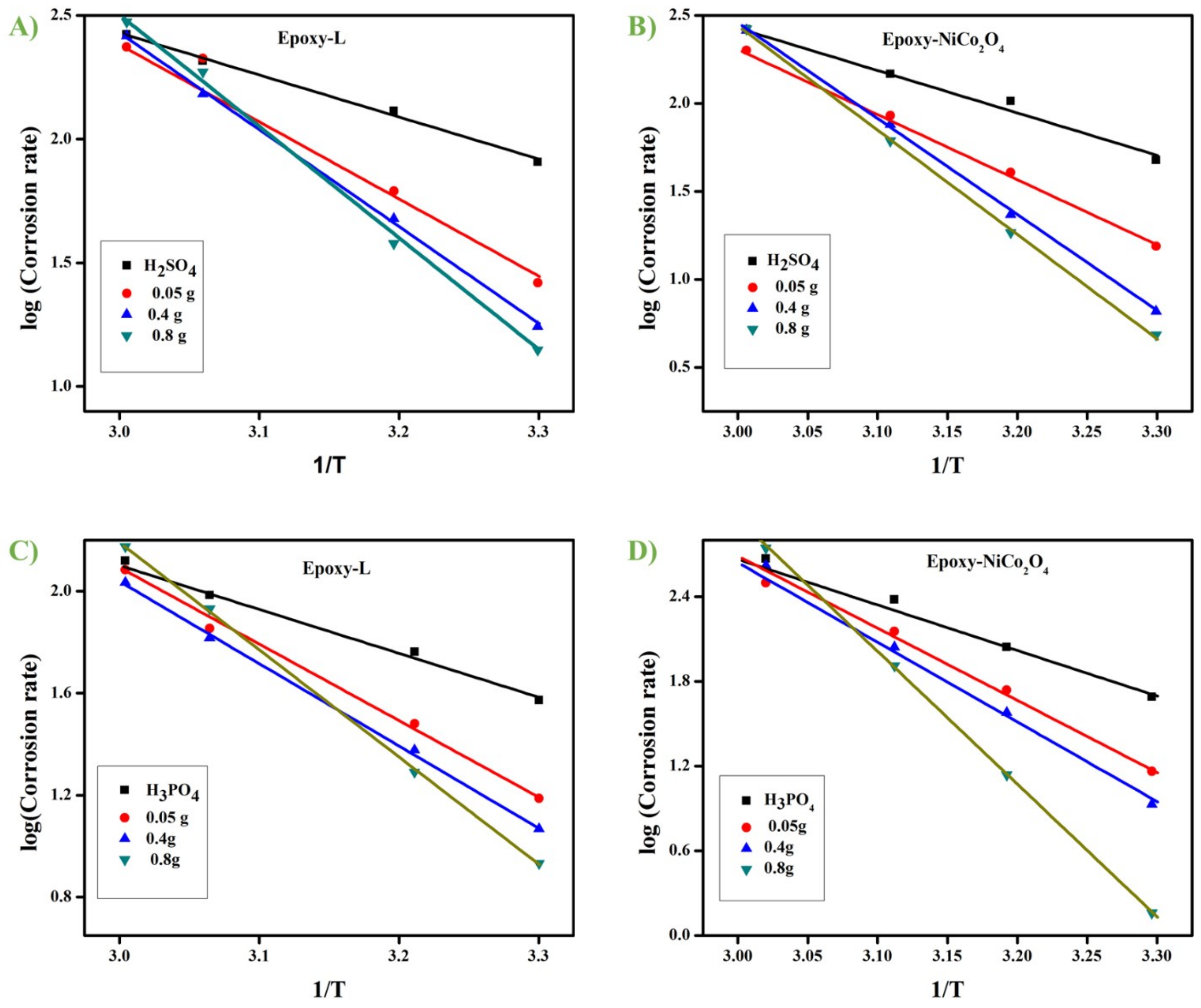

Figure S1 *Corrosion rate in mild steel: Arrhenius plots with H_2SO_4- **A)** epoxy -L and **B)** epoxy-$NiCo_2O_4$; with H_3PO_4- **C)** epoxy -L and **D)** epoxy-$NiCo_2O_4$.*

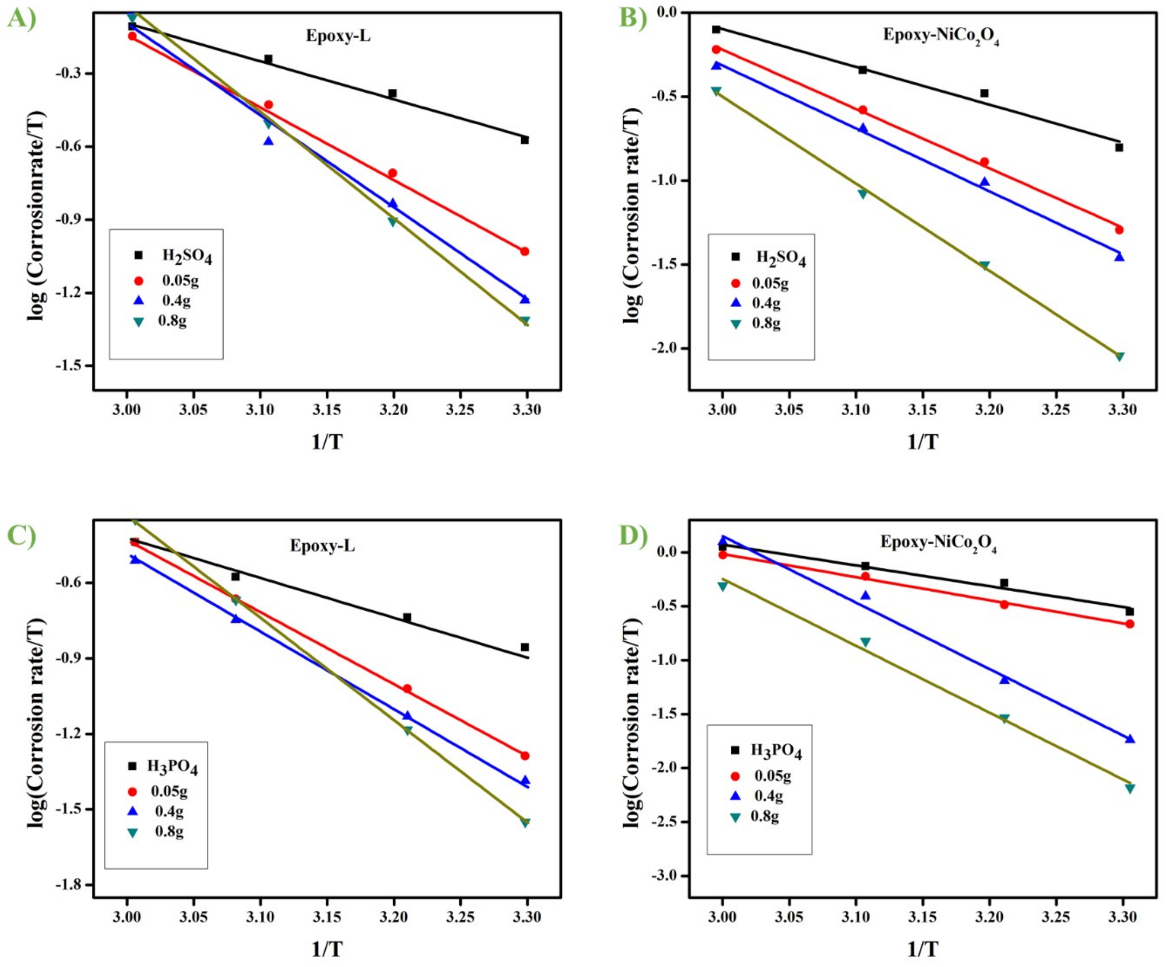

Figure S2 *Corrosion rate in mild steel: Transition plot with H_2SO_4- **A)** epoxy -L and **B)** epoxy-$NiCo_2O_4$; with H_3PO_4- **C)** epoxy -L and **D)** epoxy-$NiCo_2O_4$.*
